# Editorial: Telehealth innovations in vascular care

**DOI:** 10.3389/fsurg.2026.1788416

**Published:** 2026-01-30

**Authors:** Davide Costa, Umberto Marcello Bracale, Raffaele Serra

**Affiliations:** 1 Interuniversity Center of Phlebolymphology (CIFL), International Research and Educational Program in Clinical and Experimental Biotechnology, University “Magna Graecia” of Catanzaro, Catanzaro, Italy; 2 Department of Medical and Surgical Sciences, University “Magna Graecia” of Catanzaro, Catanzaro, Italy; 3 Department of Public Health, Federico II University Hospital, Naples, Italy

**Keywords:** innovation, telehealth, vascular care, vascular disease, vascular surgery

Telehealth has emerged as a transformative force in contemporary healthcare, reshaping how services are delivered, accessed, and experienced by patients and providers alike. In vascular care, where continuity of surveillance, prompt clinical assessment, and multidisciplinary coordination are essential, remote health solutions hold particular promise as they can expand access and improve outcomes while confronting persistent barriers such as geographic isolation and resource limitations. The Research Topic “*Telehealth Innovations in Vascular Care*” brings together original research and clinical insights that collectively illuminate the role telehealth can play across diverse aspects of vascular practice, especially in the wake of the COVID-19 pandemic and its enduring impact on care delivery.

The articles in this collection span methodological innovation, large-scale utilization patterns, procedural techniques with telehealth relevance, and comparative clinical practice, illustrating both the versatility and the ongoing challenges of integrating telehealth into vascular care pathways.

The first article, *Efficacy and safety of the integrated puncture method vs. the conventional puncture method in Peripherally Inserted Central Catheter placement for cancer patients* (Wang et al.), investigates a procedural innovation relevant to remote support of vascular access procedures. Although primarily focused on an interventional technique, its findings have implications for how telehealth can support procedural planning and training. The study's retrospective analysis of 224 patients demonstrates that an integrated puncture method achieves significantly higher first-attempt success rates, reduces procedural time, and lowers exposure risks compared to conventional puncture approaches. These results suggest that, paired with remote guidance and tele-supervision, such optimized techniques could enhance safety and efficacy in settings with limited on-site expertise.

The second contribution, *Endovascular management of a fractured dialysis catheter: a case report and review of retrieval techniques* (Lin et al.), highlights the critical role of telehealth-supported collaboration in handling rare but potentially life-threatening vascular complications. This case report describes the successful percutaneous retrieval of an intravascular catheter fragment that migrated to the right atrium. While not a telehealth intervention *per se*, the article underscores how telecommunication and digital imaging exchange can facilitate multidisciplinary decision-making, expedite remote consultation with specialists, and guide care in urgent scenarios where time and expertise are constrained.

Complementing these clinical procedural insights, the third article, *Telemedicine in Polish primary care during and after the COVID-19 crisis: a retrospective analysis of over 720,000 consultations* (Zakrzewski et al.), provides a comprehensive population-level evaluation of remote care utilization patterns. Drawing on a nationwide dataset spanning over 720,000 consultations, the authors document the dramatic rise in teleconsultations during the pandemic, peaking at nearly 80% of visits, and their stabilization at approximately 12%–15% in the post-pandemic period. The study reveals demographic disparities in telemedicine use, with older adults less likely to utilize remote visits, and modality-specific differences in clinical actions (e.g., prescriptions issued more often during teleconsultations). These findings underscore both the resilience of telehealth integration into routine care and the persistent need to address equity, technology proficiency, and infrastructure to optimize its long-term uptake in vascular and general practice settings.

The fourth article, *Clinical comparison of transfemoral vs. distal transradial access for lower extremity arteriography* (Feng et al.), delivers valuable comparative data on vascular access approaches that may be informed by remote assessment and planning. Although its primary focus is anatomical and technical, the study's outcomes regarding procedural safety and efficacy can inform virtual pre-procedure evaluations and shared decision-making between clinicians and patients in telehealth contexts. By quantifying differences in procedural metrics and complications between access routes, the article serves as a foundation for future telemedicine applications that support individualized procedural planning and remote risk stratification.

Together, the articles in this Research Topic highlight that telehealth in vascular care encompasses both direct remote interactions (e.g., teleconsultations) and broader digital health–enabled practices such as remote planning, interpretation of imaging, multidisciplinary coordination, and patient follow-up. Across all contributions, there is a clear signal that telehealth can enhance access, streamline care pathways, and support clinical decision-making when integrated purposefully within existing clinical workflows. At the same time, the collection reflects important limitations: disparities in access and utilization, the need for robust infrastructure and digital literacy, and the absence of standardized frameworks to guide clinical implementation.

Looking forward, future research should emphasize prospective evaluations of telehealth interventions tailored to specific vascular disease states, the validation of remote monitoring tools and wearable technologies for longitudinal surveillance, and the development of evidence-based guidelines that ensure safe, equitable, and effective telehealth practice. Moreover, as digital health ecosystems evolve, equitable access and user-centered design must remain central to innovation to avoid perpetuating disparities among vulnerable patient populations.

In conclusion, “*Telehealth Innovations in Vascular Care*” offers a multifaceted examination of how digital modalities are reshaping vascular practice, fostering innovation not only in remote consultation and care delivery but also in the procedural, collaborative, and system-level domains that define contemporary vascular healthcare.

